# Induction and mediation of allergy reactions by prostaglandin D2 signaling

**DOI:** 10.1186/1939-4551-8-S1-A149

**Published:** 2015-04-08

**Authors:** Masataka Nakamura

**Affiliations:** 1 Tokyo Medical and Dental University, Japan

## Background

The present study examined the functional roles of the prostaglandin D2 (PGD2) receptors in allergic and immune reactions in mouse. We previously cloned the gene for a G protein-coupled receptor named CRTH2 (chemoattractant receptor-homologous molecule expressed on Th2) and revealed that CRTH2 functions as a receptor for PGD2 with preferential expression on Th2 cells, eosinophils and basophils in human. PGD2 is an arachidonic acid metabolite that has long been implicated in allergic and immune reactions. PGD2 exerts its activities by binding to PGD2 receptors (DP and CRTH2) on target cells.

## Methods

BALB/c and C57BL/6 mice lacking the functional CRTH2 gene were generated (CRTH2 KO). DP deficient mice were kindly given by Dr. Narumiya of Kyoto University (DP KO). Cross-breeding between two mutant mice generated CRTH2- and DP-deficient double knockout mice (CRTH2/DP KO). Inflammatory reactions in mouse models of allergic cutaneous disorders and pollinosis were examined. In addition, Th1 and Th2 inflammatory reactions were induced by injection with complete Freund's adjuvant (CFA) and infection with Nippostrongylus brasiliensis (Nb), respectively. Ramatroban, a selective CRTH2 antagonist, was used.

## Results

Ear-swelling responses induced by hapten-specific IgE were less pronounced in CRTH2 KO with reduction in infiltration of eosinophils and production of chemokines. DP KO exhibited increased inflammatory reactions than wild-type mice. Interestingly CRTH2/DP KO showed exacerbated responses like DP KO. Similar exacerbation was seen with mice lacking the hematopoietic-type PGD synthase (H-PGDS) gene. CRTH2 KO also exhibited relief from frequent sneezing and rubbing induced by pollen administration with reduction in antigen-specific IgE/IgG levels, IL-4 production and nasal eosinophilia. Allergic responses of IgE production, Th2 cytokine production and eosinophilia caused by Nb infection were also reduced in CRTH2 KO than wild type mice. CFA-mediated IFN-g production however was significantly enhanced in CRTH2 KO. These pathophysiological phenotypes observed with CRTH2 KO were close to those seen in mice administrated with Ramatroban.

## Conclusions

PGD2 delivers signals for induction and mediation of allergic reactions in a PGD2 receptor-dependent manner.

